# Identification of Phosphorylated Amino Acids in Human TNRC6A C-Terminal Region and Their Effects on the Interaction with the CCR4-NOT Complex

**DOI:** 10.3390/genes12020271

**Published:** 2021-02-13

**Authors:** Fusako Munakata, Masataka Suzawa, Kumiko Ui-Tei

**Affiliations:** 1 Department of Biological Sciences, Graduate School of Science, The University of Tokyo, Tokyo 113-0033, Japan; m-f123@g.ecc.u-tokyo.ac.jp (F.M.); suzawa@g.ecc.u-tokyo.ac.jp (M.S.); 2 Department of Computational Biology and Medical Sciences, Graduate School of Frontier Sciences, The University of Tokyo, Chiba 277-8561, Japan

**Keywords:** TNRC6A, GW182 family protein, phosphorylation, RNA silencing, CCR4-NOT, protein–protein interaction

## Abstract

Human GW182 family proteins have Argonaute (AGO)-binding domains in their N-terminal regions and silencing domains, which interact with RNA silencing-related proteins, in their C-terminal regions. Thus, they function as scaffold proteins between the AGO protein and RNA silencing-related proteins, such as carbon catabolite repressor4-negative on TATA (CCR4-NOT) or poly(A)-binding protein (PABP). Our mass spectrometry analysis and the phosphorylation data registered in PhosphoSitePlus, a post-translational modification database, suggested that the C-terminal region of a human GW182 family protein, TNRC6A, has at least four possible phosphorylation sites, which are located near the region interacting with the CCR4-NOT complex. Among them, two serine residues at amino acid positions 1332 and 1346 (S1332 and S1346) were certainly phosphorylated in human HeLa cells, but other two serine residues (S1616 and S1691) were not phosphorylated. Furthermore, it was revealed that the phosphorylation patterns of TNRC6A affect the interaction with the CCR4-NOT complex. When S1332 and S1346 were dephosphorylated, the interactions of TNRC6A with the CCR4-NOT complex were enhanced, and when S1616 and S1691 were phosphorylated, such interaction was suppressed. Thus, phosphorylation of TNRC6A was considered to regulate the interaction with RNA silencing-related factors that may affect RNA silencing activity.

## 1. Introduction

RNA silencing is a post-transcriptional gene silencing mechanism induced by approximately 22-nucleotide-long non-coding RNA, called microRNA (miRNA), and is widely conserved in eukaryotes [1,2,3]. MiRNA is first transcribed from genomic DNA in the nucleus as a stem-loop-structured primary-miRNA (pri-miRNA). Pri-miRNA becomes a hairpin-structured precursor-miRNA (pre-miRNA) by processing the flanking region via a microprocessor complex consisting of Drosha and DiGeorge Syndrome Critical Region Gene 8 (DGCR8). Pre-miRNA is transported from the nucleus to the cytoplasm by Exportin-5 and further processed by Dicer into an miRNA duplex in the miRNA-induced silencing complex (miRISC) loading complex (RLC) [4,5]. The MiRNA duplex is loaded on Argonaute (AGO) protein in the miRISC [6,7]. One of the RNA strands is removed, and another single-stranded RNA remains as a mature miRNA on the miRISC. The miRNA binds to mRNAs that have sequence complementarities mainly to the miRNA seed region (nucleotides 2 to 8 from the 5´ terminal) in the 3´ UTRs, causing translational suppression and mRNA degradation [8].

Human has three paralogs of the GW182 family proteins (TNRC6A, TNRC6B, TNRC6C). Each of them has a glycine-tryptophan-repeat (GW)-rich AGO-binding domain in the N-terminal region [9,10,11,12,13,14,15,16,17]. In the C-terminal region of each TNRC6 protein, known as a silencing domain (SD), contains poly(A)-binding protein (PABP)-binding motif 2 (PAM2), RNA recognition motif (RRM), carbon catabolite repressor4-negative on TATA (CCR4-NOT) interaction motif, CNOT interacting motif 1 (CIM1), and CIM2 [18,19,20,21,22,23,24,25]. In the center, the ubiquitin-associated like domain (UBA), glutamine rich region (Q rich), nuclear export signal (NES), and nuclear localization signal (NLS) are observed. Thus, TNRC6 acts as a scaffold protein that links the AGO protein through its N-terminal domain to RNA silencing-related factors, such as CCR4-NOT, PABP, or PAN2-PAN3, via its C-terminal domain and suppresses translation of the target mRNAs. The CCR4-NOT complex consists of multiple proteins including the scaffold protein, CNOT1, TNRC6A-interacting CNOT9, or deadenylases (CNOT7 and CNOT8). AGO and GW182 family proteins are localized in a structure called the processing-body (P-body) in the cells. The P-body has a dynamic structure in which many mRNAs and RNases are assembled and is an intracellular organelle where mRNA is finally destructed [18,19,20,21].

**Figure 1:**
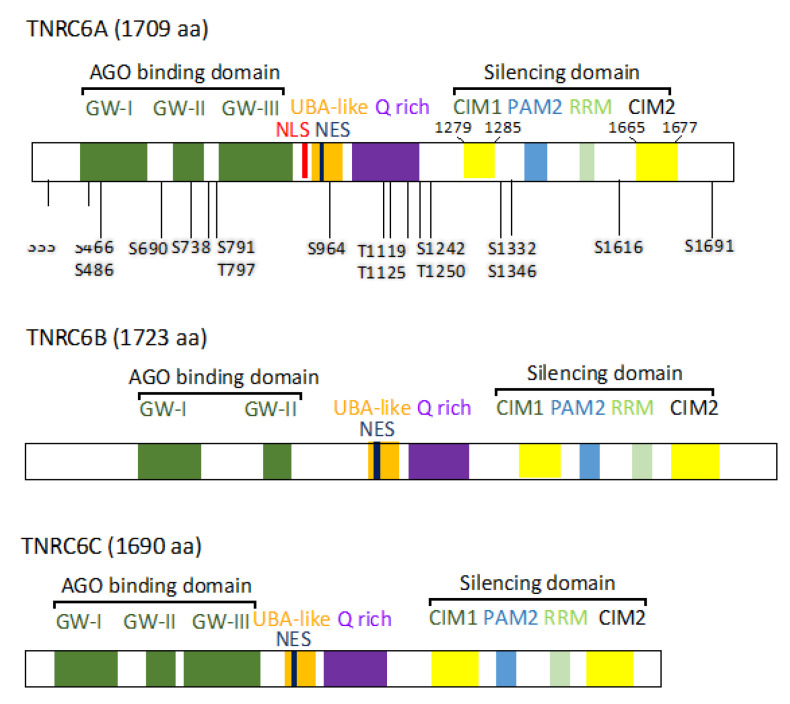
Domain structures of human TNRC6A, TNRC6B, and TNRC6C. N-terminal regions of TNRC6 family proteins are Argonaute (AGO)-binding domains, and C-terminal regions are silencing domains. QW indicates highly homologous GW/WG regions. NLS, nuclear localization signal; NES, nuclear export signal; UBA-like, ubiquitin-associated like domain; Q rich, glutamine-rich region; PAM2, poly A binding protein binding motif 2; RRM, RNA recognition motif; CIM1, carbon catabolite repressor4-negative on TATA (CCR4-NOT) interaction motif 1; CIM2, CCR4-not interaction motif 2.

Recently, it has been reported that the phosphorylation of the AGO protein stabilizes the interaction with the GW182 family proteins and promotes RNA silencing activity [22], indicating that RNA silencing may be also regulated by phosphorylation of proteins other than AGO. TNRC6A has been reported to have multiple phosphorylated residues, but their functions remain unknown [23,24,25,26]. In our previous research, 17 phosphorylation candidate sites were identified by mass spectrometry of TNRC6A (Figure 1) [27]. Among them, four candidate amino acids at the C-terminal region were detected in the vicinity of CIM1 and CIM2. It is speculated that the phosphorylation of amino acids near CIM1 and CIM2 affects the interaction of TNRC6A with the CCR4-NOT deadenylation complex. Then, we identified the amino acids that are actually phosphorylated in HeLa cells, and revealed that the phosphorylation patterns of those amino acids affect the interaction with some RNA silencing-related factors involved in the CCR4-NOT complex. 

## 2. Materials and Methods

### 2.1. Cell Culture

Human HeLa cells (human cervical-cancer-derived cells) were cultured in Dulbecco’s modified Eagle’s medium (DMEM) containing 10% heat-inactivated fetal bovine serum (FBS) at 37 °C in the presence of 5% CO_2_. The HeLa cell line was obtained from Hiroyuki Sasaki at Kyushu University.

### 2.2. Construction of Expression Constructs of Phosphorylated TNRC6A Mutants

#### 2.2.1. TNRC6A Expression Construct with Myc-GFP Tag at N-Terminus

Using pcDNA3.1-myc-GFP-TNRC6A [15], the expression constructs of the SD fragment and its phosphorylated mutants, TNRC6A-SD-CIM1 and its phosphorylated mutants, and TNRC6A-SD-CIM2 and its phosphorylated mutants, tagged with myc and GFP (Green Fluorescent Protein) at their N-terminus, were generated by amplification using primers shown in Table S1 by inverse PCR with KOD Plus (TOYOBO, Osaka, Japan). The prepared expression plasmid was transformed into *Escherichia coli* (*E. coli*) JM109 and purified using the minipreps DNA purification system (Promega, Madison, WI, USA). Their sequencing was performed to confirm that the mutation was correctly incorporated.

The constructs expressing SD fragments generated by the above method are as follows: The constructs pcDNA3.1-myc-GFP-TNRC6A-SD-CIM1-WT and pcDNA3.1-myc-GFP-TNRC6A-SD-CIM2-WT express wild-type CIM1 and CIM2 fragments, respectively. The mutant pcDNA3.1-myc-GFP-TNRC6A-SD-CIM1-S1332D expresses the CIM1 fragment with serine (S) at amino acid 1332 substituted with aspartic acid (D). The mutants pcDNA3.1-myc-GFP-TNRC6A-SD-CIM1-S1346D and pcDNA3.1-myc-GFP-TNRC6A-SD-CIM1-S1332D/S1346D were also constructed. Furthermore, pcDNA3.1-myc-GFP-TNRC6A-SD-CIM1-S1332A, pcDNA3.1-myc-GFP-TNRC6A-SD-CIM1-S1346A, and pcDNA3.1-myc-GFP-TNRC6A-SD-CIM1-S1332A/S1346A were generated. In these expression constructs, S1332 and/or S1346 were substituted with alanines (As). In addition, the CIM2 mutants, pcDNA3.1-myc-GFP-TNRC6A-SD-CIM2-S1616D, pcDNA3.1-myc-GFP-TNRC6A-SD-CIM2-CIM1-S1691D, pcDNA3.1-myc-GFP-TNRC6A-SD-CIM2-S1616D/S1691D, pcDNA3.1-myc-GFP-TNRC6A-SD-CIM2-S1616A, pcDNA3.1-myc-GFP-TNRC6A-SD-CIM2-S1691A, and pcDNA3.1-myc-GFP-TNRC6A-CIM2-SD-S1616A/S1691A, were also constructed. Furthermore, the CIM1 and CIM2 mutant, pcDNA3.1-myc-GFP-TNRC6A-CIM2-SD- S1332A/S1346A/S1616A/S1691A, was also constructed.

#### 2.2.2. TNRC6A Expression Construct with FLAG-HA-SBP Tag at N-Terminus

Using pcDNA5.1-FLAG-HA-SBP-TNRC6A [15] as a template, the expression constructs of the SD fragment with their phospho-mimic and phospho-deficient mutants tagged with FLAG-HA-SBP at their N-terminus were amplified with primers shown in Table S1 by inverse PCR with KOD Plus.

The constructs expressing SD fragments generated by the above method are as follows: pcDNA5.1-FLAG-HA-SBP(FHS)-TNRC6A-SD-WT, pcDNA5.1-FHS-TNRC6A-SD-S1332D/S1346D, pcDNA5.1-FHS-TNRC6A-SD-S1332A/S1346A, pcDNA5.1-FHS-TNRC6A-SD-S1616D/S1691D, pcDNA5.1-FHS-TNRC6A-SD-S1616A/S1691A, pcDNA5.1-FHS-TNRC6A-SD-S1332D/S1346D/S1616D/S1691D, and pcDNA5.1-FHS-TNRC6A-SD-S1332A/S1346A/S1616A/S1691A. 

#### 2.2.3. Full-Length TNRC6A Expression Construct with FHS Tag at N-Terminus

Using pcDNA5.1-FHS-TNRC6A [15] as a template, the expression constructs of full-length TNRC6A phospho-mimic and phospho-deficient mutants were amplified with primers shown in Table S1 by inverse PCR with KOD Plus.

The constructs expressing a full-length TNRC6A generated by the above method are as follows: pcDNA5.1-FHS-TNRC6A-S1332D/S1346D, pcDNA5.1-FHS-TNRC6A-S1332A/S1346A, pcDNA5.1-FHS-TNRC6A-S1616D/S1691D, and pcDNA5.1-FHS-TNRC6A-S1616A/S1691A.

### 2.3. Immunoprecipitation

Twenty-four hours before transfection, human HeLa cells were inoculated at 2.0 × 10^6^ cells/9 cm culture dish. Next, 10 μg of each of the TNRC6A expression plasmids and 30 μg of polyethylenimine (PEI) were dissolved in 1 mL of phosphate-buffered saline (PBS) and incubated at room temperature for 15 min. The mixture was added to 9 mL of DMEM (Dulbecco’s Modified Eagle’s Medium) containing 10% FBS (Fetal Bovine Serum) and replaced with fresh culture medium. The cells transfected with the construct expressing each fragment of TNRC6A were collected for immunoprecipitation at 24 h after transfection, and those transfected with a full-length TNRC6A expression construct were collected at 36 h after transfection. The cells were washed with 1 mL of PBS and centrifuged at 800× *g* for 5 min at 4 °C. To the precipitated cells, 500 μL of lysis buffer (10 mM HEPES (pH 7.9), 1.5 mM MgCl_2_, 10 mM KCl, 0.5 mM DTT, 140 mM NaCl, 1 mM EDTA, 1 mM Na_3_VO_4_, 10 mM NaF, 0.5 mM NP40, complete protease inhibitor) was added, and cells were centrifuged at 800× *g* for 5 min at 4 °C. They were incubated on ice for 10 min and centrifuged at 20× *g* for 2 min, and 450 μL of the collected supernatant was suspended with 30 μL of Sepharose beads (Sigma-Aldrich, Darmstadt, Germany) and incubated for 1 h (preclear method). After centrifugation at 800× *g* for 5 min at 4 °C, 420 μL of the supernatant was added to 20 μL of beads (Sigma-Aldrich) to which an anti-FLAG antibody was bound, and incubated for 2 h at 4 °C. After gentle centrifugation, the supernatant was removed and suspended in 500 μL of wash buffer (10 mM HEPES (pH 7.9), 1.5 mM MgCl_2_, 10 mM KCl, 0.5 mM DTT, 300 mM NaCl, 1 mM EDTA, 1 mM Na_3_VO_4_, 10 mM NaF, 0.5% NP40, complete protease inhibitor) 2 times, and lysis buffer was added. After removing the buffer, 2× SDS-PAGE sample buffer (4% SDS, 0.1 M Tris-HCL (pH 6.8), 12% 2-mercaptoethanol, 20% glycerol, 0.01% BPB (Bromophenol blue)) was added to the sample, which was then boiled for 5 min and centrifuged at 800× *g* for 1 min. The supernatant was collected and stored at −80 °C.

### 2.4. Western Blot

The cell lysate or immunoprecipitated sample boiled at 100 °C for 5 min in the SDS-PAGE sample buffer was separated on 6.5% or 12.5% denaturing polyacrylamide gel at 20 mV for 100 min. After transferring the protein to a PVDF (Polyvinylidene Fluoride) membrane (25 V constant, 10 min), blocking was performed with 5% skim milk–TBST (20 mM Tris-HCl (pH 7.5), 150 mM NaCl, 0.1% Tween 20) for 1 h. Each of the first antibodies was treated at 4 °C overnight. The antibodies used were as follows: 2000-fold-diluted anti-FLAG antibody (Sigma-Aldrich, F1804-1MG), 1000-fold-diluted anti-CNOT1 antibody (Proteintech, 14276-1-AP, Rosemont, IL, USA), 1000-fold-diluted anti-CNOT9 antibody (Proteintech, 22503-1-AP), 1:2-diluted anti-PABP antibody (Abcam, ab21060), and 3000-fold-diluted anti-β-actin antibody (Abcam, ab4074). After washing the membrane three times with TBST, each of the second antibody was treated for 1 h at room temperature. The second antibodies used were as follows: 6000-fold-diluted horseradish peroxidase (HRP)-labeled anti-rabbit IgG (GE healthcare, Hino, Tokyo, Japan) and anti-mouse IgG (GE healthcare). The membrane was washed three times with TBST, and the signals were detected with LAS4000 mini (GE Healthcare) using ECL Prime (Sigma-Aldrich) Western Blotting Detection Reagents (GE Healthcare).

### 2.5. Detection of Phosphorylated Amino Acid Residues Using Phos-Tag SDS-PAGE

pcDNA3.1-myc-GFP-TNRC6A-SD-CIM1-WT and its mutants, pcDNA3.1-myc-GFP-TNRC6A-CIM2-WT and its mutants, and pcDNA3.1-myc-GFP-TNRC6A-SD-WT and its mutants were transfected into human HeLa cells cultured in 9 cm dishes. The cells were washed with PBS, suspended in 500 μL of a phosphatase lysis buffer (10 mM HEPES (pH 7.9), 1.5 mM MgCl_2_, 10 mM KCl, 0.5 mM DTT, 140 mM NaCl, 10 mM NaF, 0.5% NP40, complete protease inhibitor), and incubated on ice for 10 min. The suspension was centrifuged at 800× *g* for 5 min at 4 °C, and the aliquot of 16 μL of the supernatant was incubated with 4 μL of calf intestine alkaline phosphatase (CIAP) (3.0 units/μL, TOYOBO) in CIAP buffer (30 mM triethanolamine (pH 7.6), 1 mM MgCl_2_, 50% glycerol) for dephosphorylation or with 4 μL of CIAP buffer as a control and incubated at 37 °C for indicated periods. Dephosphorylated and non-dephosphorylated samples were separated by electrophoresis using 7.5% SupersepTM Phos-tagR (50 μmol/L) (WAKO, Osaka, Japan). The gel was washed three times for 10 min with a transfer buffer containing 10 mM EDTA, 25 mM Tris, 192 mM glycine, and 5% SDS and washed once for 10 min with the transfer buffer to remove Mn_2_^+^, and Western blot was performed.

### 2.6. Statistical Analysis

The parametric Student’s *t*-test was performed for calculating significant differences between protein amounts involved in the independent immunoprecipitated samples.

## 3. Results

### 3.1. Interaction of C-Terminal Silencing Domain (SD) of TNRC6A with the Components Involved in the CCR4-NOT Complex

TNRC6A is a member of the human GW182 family proteins and acts as a scaffold protein that links the AGO protein to the CCR4-NOT complex in the mechanism of RNA silencing [9,10,11,12,13,14,15,16,17,28,29,30,31,32,33,34]. The CCR4-NOT complex consists of multiple proteins: CNOT1 is the largest scaffold protein in the complex, CNOT9 is involved in the interaction with TNRC6A, and CNOT7 and CNOT8 are deadenylases [35]. Furthermore, CNOT9 has a tryptophan pocket and directly binds to TNRC6A via tryptophan residues in the SD to regulate RNA silencing activity [28,31,33,34,36,37]. TNRC6A is estimated to have approximately seven tryptophan residues required for interaction with the CCR4-NOT complex, but it is unknown which tryptophan residues contribute to the interaction [31,37]. The tryptophan residues in TNRC6A involved in the interaction with the CCR4-NOT complex are considered to localize in two regions from amino acids 1279 to 1285, called CCR4-NOT interaction motif 1 (CIM1), and from 1665 to 1677, called CIM2 (Figure 1) [38].

**Figure 2:**
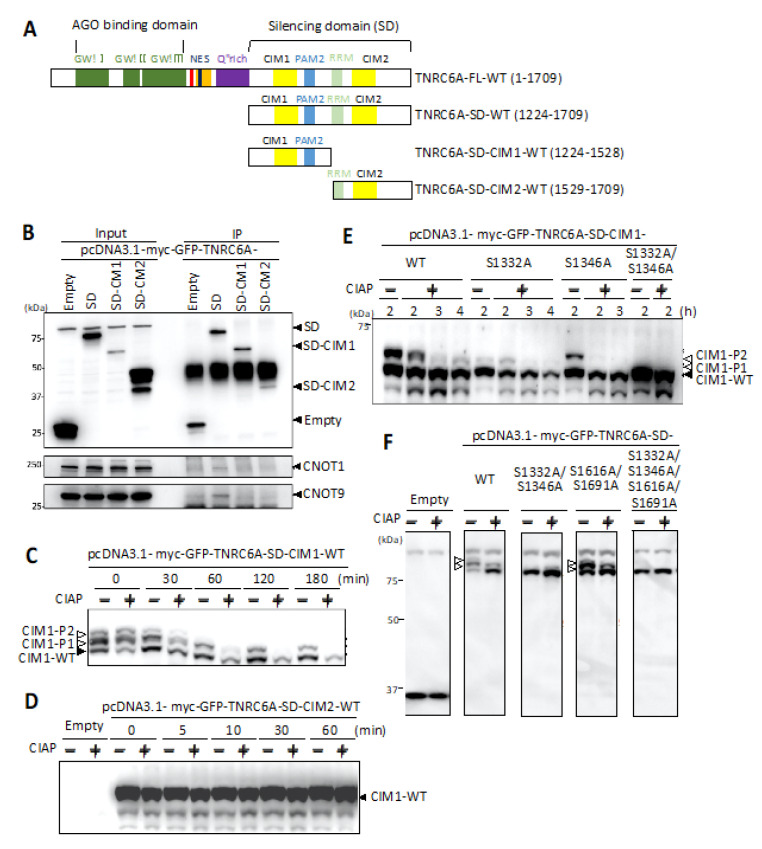
Interaction between the TNRC6A silencing domain (SD) and CNOT1/CNOT9 and identification of phosphorylated amino acids in TNRC6A-SD. (**A**) Domain structures of TNRC6A and fragments of the SD used for analysis of phosphorylation. (**B**) Cell lysates of HeLa cells expressing GFP-tagged TNRC6A-SD, TNRC6A-SD-CIM1, or TNRC6A-SD-CIM2 protein were immunoprecipitated with anti-GFP antibody, and CNOT1 and CNOT9 were detected using anti-CNOT1 and anti-CNOT9 antibody, respectively. (**C**) Phosphorylation patterns of the TNRC6A-SD-CIM1 fragment with and without phosphatase (calf intestine alkaline phosphatase (CIAP)) treatment for 0, 30, 60, 120, or 180 min were detected by the Phos-tag gel shift assay. (**D**) Phosphorylation patterns of the TNRC6A-SD-CIM2 fragment with and without CIAP treatment for 0, 5, 10, 30, or 60 min were detected by the Phos-tag gel shift assay. (**E**) Phosphorylation patterns of the TNRC6A-SD-CIM1 fragments with no mutation, S1332A, S1346A, or S1332A/S1346A with and without CIAP treatments for 2, 3, or 4 h were detected by the Phos-tag gel shift assay. (**F**) Phosphorylation patterns of the TNRC6A-SD fragments with no mutation, S1332A/S1346A, S1616A/S1691A, or S1332A/S1346A/S1616A/S1691A with and without CIAP treatment for 2 h were detected by the Phos-tag gel shift assay.

At first, to investigate whether the C-terminal region of TNRC6A interacts with the CCR4-NOT complex, constructs expressing the fragment from amino acids 1224 to 1709 corresponding to the entire SD region and the fragment containing CIM1 (amino acids 1224–1528) or that containing CIM2 (amino acids 1529–1709) were generated (Figure 2A). Each of these expression constructs was transfected into human HeLa cells, and immunoprecipitation with anti-GFP antibody was performed. The immunoprecipitates were analyzed by Western blot using anti-CNOT1 or anti-CNOT9 antibody (Figure 2B), since CNOT1 and CNOT9 are typical components of the CCR4-NOT complex. It was found that CNOT1 and CNOT9 were immunoprecipitated with the SD fragment of the TNRC6A protein (TNRC6A-SD). However, the immunoprecipitated CNOT1 and CNOT9 proteins were not observed when either fragment of TNRC6A-SD-CIM1 or TNRC6A-SD-CIM2 was expressed, suggesting that both TNRC6A-SD-CIM1 and TNRC6A-SD-CIM2 regions are necessary for CNOT1 and CNOT9 interactions, respectively. Based on these results, the TNRC6A-SD fragment was used for experiments for analyzing interactions with proteins in the CCR4-NOT complex. 

### 3.2. Identification of Phosphorylated Regions in the SD of TNRC6A

TNRC6A is known to have multiple phosphorylated residues, but their functions are unknown. In a post-translational modification database called PhosphoSitePlus (https://www.phosphosite.org/homeAction.action (accessed on 8 February 2021)), multiple candidate sites of phosphorylation in TNRC6A were registered (Table 1).

Furthermore, we identified a total of 17 phosphorylated amino acids by mass spectrometry analysis of TNRC6A using HeLa cells [Suzawa et al. in preparation] (Figure 1 and Table 2). Among them, we focused on four candidate amino acids (S1332, S1346, S1616, and S1691), which were identified as phosphorylated amino acids in the SD of TNRC6A in both PhosphoSitePlus and our mass spectrometry analysis, since the phosphorylation in the SD is expected to regulate interaction with RNA silencing-related proteins to promote RNA silencing.

Candidate amino acids of phosphorylation in the SD were positioned near CIM1 and CIM2: serines at 1332 (S1332) and 1346 (S1346) resided between CIM1 and PAM2 (Figure 1), and S1616 and S1691 were located on both sides of CIM2. To identify true phosphorylated amino acids among these serine residues, expression constructs of CIM1, pcDNA3.1-myc-GFP-TNRC6A-SD-CIM1, and of CIM2, pcDNA3.1-myc-GFP-TNRC6A-SD-CIM2, were constructed. These expression constructs were transfected into HeLa cells. After 24 h, the cells were suspended in lysis buffer, and the supernatant was collected by centrifugation. The cell supernatant was treated with or without phosphatase, CIAP, for 0, 30, 60, 120, and 180 min at 37 °C and separated by SDS-PAGE using Phos-tag (WAKO). Phos-tag SDS-PAGE is a technique that can separate proteins phosphorylated from non-phosphorylated proteins according to the phosphorylation levels by electrophoresis (Phos-tag gel shift assay). After electrophoresis, Western blot was performed using an anti-GFP antibody, and changes in phosphorylation patterns according to the indicated periods were analyzed (Figure 2C). Three bands were observed in the lanes at 0 min in both non-dephosphorylated and dephosphorylated lysates of the cells transfected with pcDNA3.1-myc-GFP-TNRC6A-SD-CIM1-WT. The lowest band, named CIM1-WT, was considered to be a TNRC6A-SD-CIM1-WT fragment that was not phosphorylated. The upper shifted two bands were considered to be phosphorylated TNRC6A-SD-CIM1, which were named CIM1-P1 and CIM1-P2, respectively. 

At 30 min, three bands similar to those observed at 0 min were observed in the lane of the cell lysate not dephosphorylated, whereas CIM1-P2 had disappeared in the dephosphorylated cell lysate. At 60 min, CIM1-P2 disappeared even without dephosphorylation, so phosphorylation near CIM1 was not only dephosphorylated by CIAP treatment but also gradually dephosphorylated with time without CIAP treatment. In the sample subjected to the dephosphorylation treatment for 60 min, almost all two bands of both CIM1-P1 and CIM1-P2 disappeared. This suggests that these two bands were shifted by phosphorylation, and that phosphorylation actually occurred in at least two amino acids near the CIM1 region of the SD in HeLa cells. However, the changes in the phosphorylation pattern in the cells transfected with pcDNA3.1-myc-GFP-TNRC6A-SD-CIM2 were not observed after the treatment with CIAP for 0, 5, 10, 30, and 60 min at 37 °C (Figure 2D). The result suggests that phosphorylation does not occur in the normal condition near the CIM2 region in HeLa cells.

### 3.3. Identification of Phosphorylated Amino Acids in the Vicinity of the CIM1 Region

The phosphorylated region was identified in the vicinity of the CIM1 region. Common phosphorylated candidates of amino acids identified by both our mass spectrometry analysis and PhosphoSitePlus were S1332 and S1346 downstream of CIM1 (Table 2). To verify that these amino acids are actually phosphorylated, expression constructs with mutated amino acids were constructed. In pcDNA3.1-myc-GFP-TNRC6A-SD-CIM1-S1332A, S1332 of the TNRC6A-SD-CIM1 fragment was replaced with alanine, and in pcDNA3.1-myc-GFP-TNRC6A-SD-CIM1-S1346A, S1346 was replaced with alanine. Furthermore, both S1332 and S1346 were simultaneously substituted with alanines in pcDNA3.1-myc-GFP-TNRC6A-SD-CIM1-S1332A/S1346A. Each of these expression constructs was transfected into HeLa cells, and their phosphorylation patterns were examined after dephosphorylation by CIAP using the Phos-tag gel shift assay (Figure 2E). Multiple bands were observed in the cells transfected with pcDNA3.1-myc-GFP-TNRC6A-SD-CIM1-WT; but CIM1-P1 and CIM1-P2, which are upshifted bands, disappeared after 3 h. On the other hand, in the case of pcDNA3.1-myc-GFP-TNRC6A-SD-CIM1-S1332A transfection, the shift of a single band of CIM1-P1 was observed at 0 h, and the band disappeared after CIAP treatment. From this result, it was inferred that S1332 is one of the actually phosphorylated amino acids. Furthermore, in the case of pcDNA3.1-myc-GFP-TNRC6A-SD-CIM1-S1346A transfection, a single band was also observed at a different position (CIM1-P2) from CIM1-P1. This band disappeared after CIAP treatment. This suggested that S1346 is another candidate amino acid of phosphorylation. Furthermore, in the double mutant of S1332 and S1346, pcDNA3.1-myc-GFP-TNRC6A-SD-CIM1-S1332A/S1346A, no upshifted band was observed even when they were not dephosphorylated. These results strongly suggested that at least S1332 and S1346 are actually phosphorylated amino acids in HeLa cells.

To clarify that S1332 and S1346 are the only phosphorylated amino acids in the SD region, the Phos-tag gel shift assay was performed using the SD fragment of TNRC6A (Figure 2F). We generated expression constructs of mutated pcDNA3.1-myc-GFP-TNRC6A-SD series: S1332 and S1346 were substituted with alanines in pcDNA3.1-myc-GFP-TNRC6A-SD-S1332A/S1346A, S1616 and S1691 were substituted with alanines in TNRC6A-SD-S1616A/S1691A, and all of the four serines were substituted in pcDNA3.1-myc-GFP-TNRC6A-SD-S1332A/S1346A/S1616A/S1691A. Each of them was transfected into HeLa cells, the dephosphorylation reaction by CIAP was performed for 2 h after 24 h, and changes in the phosphorylation patterns were investigated by the Phos-tag gel shift assay. As a result, three bands were observed in the cells transfected with pcDNA3.1-myc-GFP-TNRC6A-SD-WT (Figure 2F), but the expression of two of them decreased after CIAP treatment for 2 h. A similar result was observed in the cells transfected with pcDNA3.1-myc-GFP-TNRC6A-SD-S1616A/S1691A. Three bands were also observed without CIAP treatment, but the upper two of them decreased after CIAP treatment. On the other hand, no band shift was observed in the nonphosphorylated sample of pcDNA3.1-myc-GFP-TNRC6A-SD-S1332A/S1346A. From these results, it was inferred that S1332 and S1346 are actually phosphorylated amino acids in the TNRC6A-SD in HeLa cells.

### 3.4. Effect of Phosphorylation in the SD Fragment on the Interaction with RNA Silencing-Related Factors

**Figure 3:**
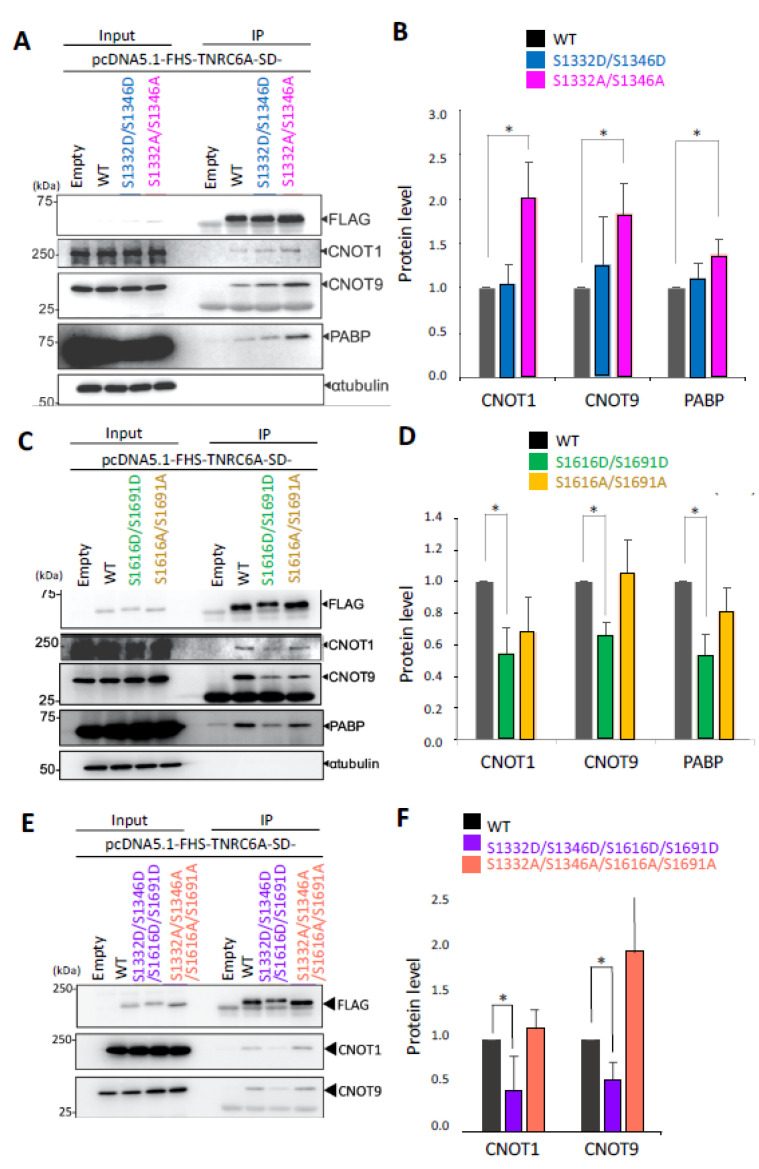
Effect of phosphorylation of the TNRC6A-SD fragment on the interaction with RNA silencing-related factors. (**A**) Immunoprecipitation using HeLa cells expressing the FLAG-HA-SBP (FHS)-tagged WT TNRC6A-SD fragment or the fragment with S1332D/S1346D or S1332A/S1346A was performed using anti-FLAG antibody. CNOT1, CNOT9, and poly(A)-binding protein (PABP) proteins were detected using anti-CNOT1, anti-CNOT9, and anti-PABP antibodies, respectively. α-tubulin was used as a negative control. (**B**) The signal intensities of CNOT1, CNOT9, and PABP proteins were measured using ImageJ, and quantified by dividing the signal intensities by anti-FLAG antibody. The data were averaged from three independent experiments, and the bar indicates the standard deviation. (**C**) Immunoprecipitation using the cells expressing the FHS-tagged WT TNRC6A-SD fragment or the fragment with S1616D/S1691D or S1616A/S1691A was performed, and CNOT1, CNOT9, and PABP proteins were detected. (**D**) The signal intensities of CNOT1, CNOT9, and PABP proteins were measured, and quantified by dividing the signal intensities by anti-FLAG antibody. The data were averaged from three independent experiments, and the bar indicates the standard deviation. (**E**) Immunoprecipitation using the cells expressing the FHS-tagged WT TNRC6A-SD fragment or the fragment with S1332D/S1346D/S1616D/S1691D or S1332A/S1346A/S1616A/S1691A was performed, and CNOT1, CNOT9, and PABP proteins were detected. (**F**) The signal intensities of CNOT1, CNOT9, and PABP proteins were measured, and quantified by dividing the signal intensities by anti-FLAG antibody. The data were averaged from three to four independent experiments. Each Western blot’s data are shown in Figure S1. * *p* < 0.05 (Student’s *t*-test).

The effect of phosphorylation in the SD region of TNRC6A on interaction with RNA silencing-related factors, such as the components of the CCR4-NOT complex or PABP, was investigated. Since S1332 and S1346 downstream of the CIM1 region were identified as phosphorylated amino acids in HeLa cells by the Phos-tag gel shift assay, a phospho-mimic mutant (pcDNA5.1-FHS-TNRC6A-SD-S1332D/S1346D) and a phospho-deficient mutant (pcDNA5.1-FHS-TNRC6A-SD-S1332A/S1346A) were constructed. Each of them was transfected into HeLa cells, and immunoprecipitation was performed using anti-FLAG antibody. Then, Western blot using anti-CNOT1, anti-CNOT9, and anti-PABP antibodies was performed, and a typical result is shown in Figure 3A. The signal intensities of proteins immunoprecipitated with each antibody were measured and quantified by dividing the corresponding signal intensities detected by anti-FLAG antibody (Figure 3B). As a result, it was revealed that higher amounts of CNOT1, CNOT9, and PABP proteins were immunoprecipitated with the TNRC6A-SD-S1332A/S1346A fragment compared to TNRC6A-SD-WT. These results suggest that two serine residues close to the CIM1 region may have a stronger interaction with these RNA silencing-related factors under non-phosphorylated conditions.

Phosphorylation of S1616 and S1691 in the vicinity of the CIM2 region could not be confirmed in the Phos-tag gel shift assay using TNRC6A-SD-CIM2 in normal HeLa cells (Figure 2D), so phosphorylation does not occur under normal conditions in the cells and may occur only under specific conditions. To understand whether phosphorylation under specific conditions may act to control the interaction with RNA silencing-related factors, a phospho-mimic mutant (pcDNA5.1-FHS-TNRC6A-SD-S1616D/S1691D) and a phospho-deficient mutant (pcDNA5.1-FHS-TNRC6A-SD-S1616A/S1691A) of S1616 and S1691 on both sides of the CIM2 region were generated. Each of the pcDNA5.1-FHS-TNRC6A-SD-S1616D/S1691D, pcDNA5.1-FHS-TNRC6A-SD-S1616A/S1691A, and pcDNA5.1-FHS-TNRC6A-SD-WT was transfected into HeLa cells, and immunoprecipitation was performed using anti-FLAG antibody. Then, Western blot was carried out using anti-CNOT1, anti-CNOT9, or anti-PABP antibody to examine the interaction with each protein (Figure 3C,D). A typical result of Western blot is shown in Figure 3C, and the averaged signal intensities of proteins immunoprecipitated with each antibody in three independent experiments are measured and quantified in Figure 3D. The interaction of TNRC6A-SD-S1616D/S1691D with CNOT1, CNOT9, or PABP was significantly attenuated compared to TNRC6A-SD-WT. On the other hand, TNRC6A-SD-S1616A/S1691A did not change the amounts of interacting CNOT1, CNOT9, or PABP protein compared to TNRC6A-SD-WT.

In the vicinity of the CIM2 region, no actual phosphorylation of S1616 and S1691 could be observed, so it was considered that these two sites may not be normally phosphorylated, at least in HeLa cells. However, by substituting these two positions with aspartic acids, the interaction with the RNA silencing factor was suppressed. Phosphorylation in S1616 and S1691 could not be detected in HeLa cells used in this study, but they may be phosphorylated under different conditions or in different cell types. Then, when S1616 and S1691 are phosphorylated under specific conditions, it is possible that the interaction with RNA silencing-related factors might be attenuated. Alternatively, the phosphorylation of S1616 and S1691 may be induced by an autophosphorylation reaction through other regions not included in the TNRC6A-SD.

Phosphorylation at S1616 and S1691 were not confirmed by the gel shift assay in this experiment, although S1332 and S1346 in the CIM1 region were clearly confirmed to be phosphorylated in the Phos-tag gel shift assay. The phospho-mimic mutant (TNRC6A-SD-S1332D/S1346D/S1616D/S1691D), in which four amino acids were simultaneously substituted with aspartic acids, and the phospho-deficient mutant (TNRC6A-SD-S1332A/S1346A/S1616A/S1691A), in which four sites were simultaneously substituted with alanines, were generated. Each of these constructs was transfected into HeLa cells, immunoprecipitation was performed using anti-FLAG antibody, and CNOT1, CNOT9, or PABP was detected by each antibody (Figure 3E,F). The interaction of TNRC6A-SD-S1332D/S1346D/S1616D/S1691D with CNOT1 or CNOT9 was strongly attenuated. On the other hand, the interaction of TNRC6A-SD-S1332A/S1346A/S1616A/S1691A with CNOT1 and CNOT9 was enhanced. Therefore, it is possible that these four serine residues regulate the interaction with RNA silencing-related factors, such as CNOT1, CNOT9, and PABP, by phosphorylation.

### 3.5. Effect of Phosphorylation of the SD in Full-Length (FL) TNRC6A on Interaction with RNA Silencing-Related Factors

**Figure 4:**
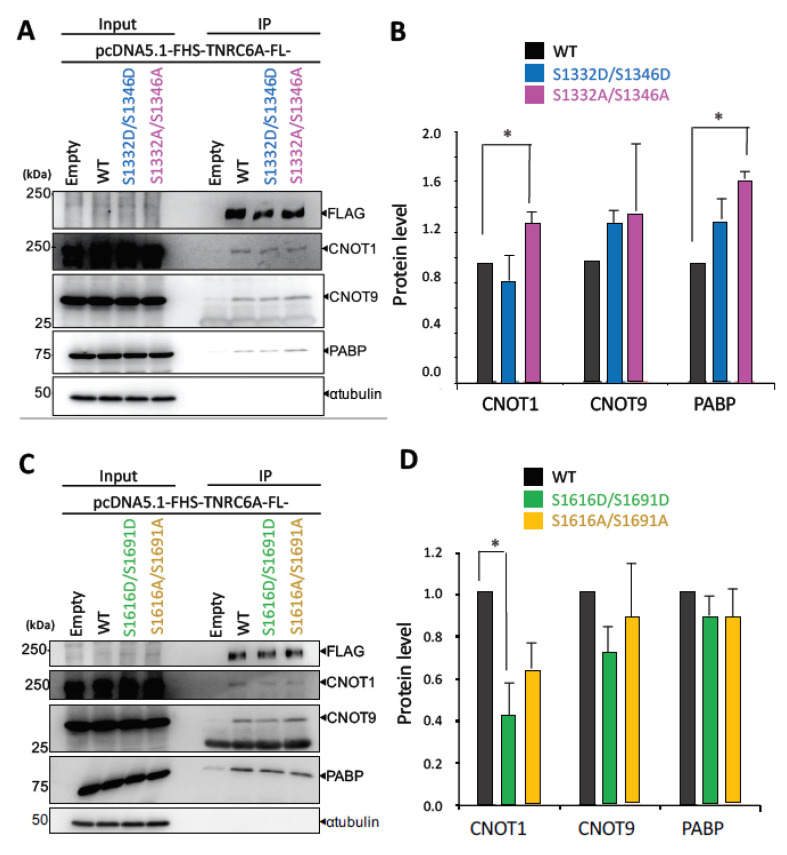
Effect of phosphorylation of full-length (FL) TNRC6A on the interaction with RNA silencing-related factors. (**A**) Immunoprecipitation using HeLa cells expressing FLAG-HA-SBP (FHS)-tagged WT TNRC6A-FL or TNRC6A with S1332D/S1346D or S1332A/S1346A was performed using anti-FLAG antibody. CNOT1, CNOT9, and PABP proteins were detected using anti-CNOT1, anti-CNOT9, and anti-PABP antibodies, respectively. α-tubulin was used as a negative control. (**B**) The signal intensities of CNOT1, CNOT9, and PABP proteins shown in (**A**) were measured using ImageJ, and quantified by dividing the signal intensities by anti-FLAG antibody. (**C**) Immunoprecipitation using the cells expressing FHS-tagged WT TNRC6A FL or TNRC6A with S1616D/S1691D or S1616A/S1691A was performed, and CNOT1, CNOT9, and PABP proteins were detected. (**D**) The signal intensities of CNOT1, CNOT9, and PABP proteins shown in (**C**) were measured, and quantified by dividing the signal intensities by anti-FLAG antibody. The data shown in (**B**) and (**D**) were averaged from two independent experiments. Each Western blot’s data are shown in Figure S2. * *p* < 0.05 (Student’s *t*-test).

The effect of phosphorylation in the case of full-length TNRC6A (FL-TNRC6A) on the interaction with RNA silencing-related factors was confirmed by Western blot. The expression construct of a phospho-mimic mutant of FL-TNRC6A (pcDNA5.1-FHS-FL-TNRC6A-S1332D/S1346D) and that of a phospho-deficient mutant of full-length TNRC6A (pcDNA5.1-FHS-FL-TNRC6A-S1332A/S1346A) were transfected into HeLa cells, and Western blot using anti-CNOT1, anti-CNOT9, or anti-PABP antibody was performed. FL-TNRC6A-S1332D/S1346D showed almost no effects on the interaction with CNOT1, CNOT9, and PABP. However, FL-TNRC6A-S1332A/S1346A enhanced the interaction with CNOT1 and PABP (Figure 4A,B). Furthermore, the transfection of the phospho-mimic mutant of FL-TNRC6A (TNRC6A-S1616D/S1691D) reduced the interaction with CNOT1, and the phospho-deficient mutant of FL-TNRC6A (pcDNA5.1-FHS-FL-TNRC6A-S1616A/S1691A) showed almost no effects on the interaction with CNOT9 and PABP (Figure 4C,D). These results were almost consistent with the results using the SD fragment (TNRC6A-SD) (Figure 3), but the effects were weaker.

## 4. Discussion

TNRC6A has the important function of regulating RNA silencing activity as a scaffold protein that interacts with the AGO protein and the RNA silencing-related factors. In the RNA silencing pathway, TNRC6A acts as one of the factors constituting the miRISC, in which the expression of mRNAs having partial sequence complementarities to the miRNA are suppressed by degradation of mRNAs. It is known that some RNA silencing-related factors included in the miRISC undergo post-translational modifications that regulate their activities, stabilities, or localizations [39,40,41,42]. In terms of phosphorylation, it is known that the interaction with the GW182 family proteins is enhanced when the serine at 387 of the AGO2 protein is phosphorylated [39,42] and phosphorylation of the tyrosine at 529 inhibits localization in the P-body and targets mRNA cleavage activity [41]. It has been suggested that the human GW182 family protein, TNRC6A, is a phosphorylated protein [18]. However, although the presence of multiple phosphorylated residues has been pointed out, nothing or little is known about the function of the phosphorylated amino acids. Previously, Huang et al. reported a total of 34 full-length TNRC6A phosphorylation candidate sites by computer analysis [43]. When all of the candidate sites were simultaneously substituted with alanines, the interaction of TNRC6A with PABP was enhanced through the PAM2 motif [43]. However, its interaction with the CCR4-NOT deadenylation complex, which contains a factor that causes deadenylation, was unknown.

**Figure 5:**
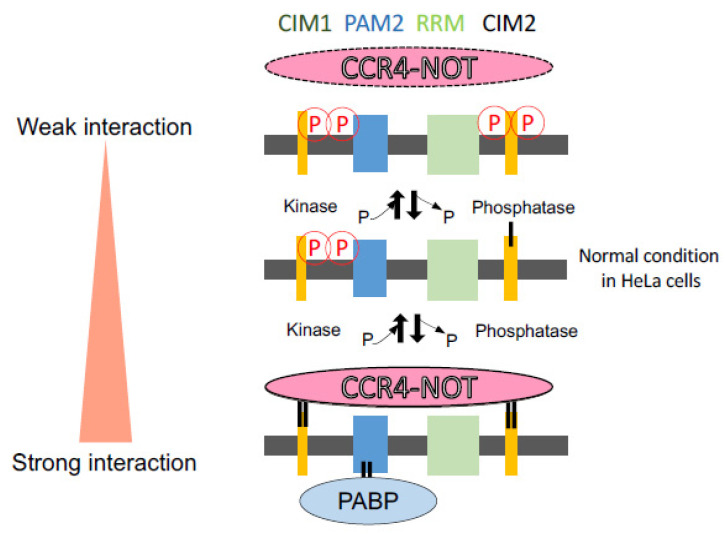
Possible regulation by the phosphorylation in the SD region of TNRC6A of the interaction with RNA silencing related-factors. In HeLa cells, two serine residues (S1332, S1346) close to CIM1 were phosphorylated, and two serine residues (S1616, S1691) close to CIM2 were not phosphorylated. However, dephosphorylation of S1332 and S1346 enhanced the interaction with CNOT1, CNOT9, and PABP, and phosphorylation of S1616 and S1691 repressed their interactions. Thus, phosphorylation of these serine residues may inhibit the interaction with RNA silencing-related factors, and their dephosphorylation may enhance their interactions. Such interactions are considered to regulate RNA silencing activities.

In this study, we focused on the SD region that interacts with the CCR4-NOT deadenylation complex in RNA silencing. We identified phosphorylated and non-phosphorylated amino acids in human HeLa cells (Figure 2) and analyzed their functions in the interaction with RNA silencing-related factors (Figure 3 and Figure 4). We focused on four amino acids (S1332, S1346, S1616, and S1691) that are registered in the PhosphoSitePlus phosphorylation database and also identified the phosphorylation sites in our mass spectrometry analysis. Their phospho-deficient mutants revealed by Phos-tag SDS-PAGE that S1332 and S1346 in the vicinity of CIM1 of the TNRC6A-SD were certainly phosphorylated, at least in HeLa cells, but S1616 and S1691 were not. Furthermore, the immunoprecipitation experiments suggested that wild-type (TNRC6A-SD-WT or TNRC6A-FL-WT) and phospho-mimic mutants of S1332 and S1346 (TNRC6A-SD-S1332D/S1346D or TNRC6A-FL-S1332D/S1346D) interact with RNA silencing-related factors, CNOT1, CNOT9, and PABP, at similar levels, but such interaction was enhanced by the phospho-deficient mutants (TNRC6A-SD-S1332A/S1346A or TNRC6A-FL-S1332A/S1346A), suggesting that S1332 and S1346 are phosphorylated in HeLa cells under normal conditions (Figure 5). However, wild-type (TNRC6A-SD-WT or TNRC6A-FL-WT) and phospho-deficient mutants of S1616 and S1691 (TNRC6A-SD-S1616A/S11691A or TNRC6A-FL- S1616A/S11691A) interact with RNA silencing-related factors at similar levels (Figure 3 and Figure 4), but such interaction was attenuated by the phospho-mimic mutants (TNRC6A-SD-S1616D/S11691D or TNRC6A-FL- S1616D/S11691D), suggesting that S1616 and S1691 are not phosphorylated in HeLa cells under normal conditions (Figure 5). These results suggest that the phosphorylation may be regulated by different types of phosphorylation enzymes and/or phosphatases in S1332/S1346 and S1616/S1692, respectively.

One hypothesis that may explain our results may be that TNRC6A phosphorylation plays the role of a switch in the interaction with RNA silencing-related factors (Figure 5). The interactions with RNA silencing-related factors are stronger for phospho-deficient alanine-substituted mutants compared to phospho-mimic aspartic-acid-substituted mutants in both S1332/S1346 and S1616/S1692 sites, at least when TNRC6A-SD fragments are used. It has been reported that tryptophan residues present in the SD region are important for interaction with the CCR4-NOT complex [28,31,33,34,36,37]. CNOT9 has two tryptophan pockets for interaction with tryptophan residues of the TNRC6A-SD region, and it is known that the insides of the pockets are highly hydrophobic [37]. The interaction of the alanine-substituted TNRC6A mutants with the CCR4-NOT deadenylation complex was enhanced, probably because it is easy for a nonpolar amino acid to enter the tryptophan pocket inside CNOT9, when the amino acids close to the tryptophan are nonpolar like alanine. Thus, the phosphorylation patterns may be regulated by the phased manner in TNRC6A by various environmental stimulations. Then, the replicated Western blot patterns were slightly flexible (Figure 3 and Figure 4 and Figures S1 and S2). Then, such phosphorylation patterns function to control the interacting activities with RNA silencing-related factors, probably resulting in the regulation of RNA silencing activity. We performed experiments to measure the RNA silencing activities in HeLa cells transfected with pcDNA5.1-FHS-TNRC6A-FL-S1332D/S1346D/S1616D/S1691D or pcDNA5.1-FHS-TNRC6A-FL-S1332A/S1346A/S1616A/S1691A (Figure S3). However, almost no significant changes in RNA silencing activities were observed, probably because the endogenous TNRC6A showed flexible phosphorylation patterns. Therefore, it may be necessary to generate TNRC6A-knockout cells to clarify the function of phosphorylation in the cells.

## Figures and Tables

**Table 1:** Phosphorylation sites of TNRC6A registered in PhosphoSitePlus.

Amino Acid Positions	Amino Acid	Sequence	References (>5)
486	S	TAWDTETsPRGERKT	9
690	S	DSSKPVSsPDWNKQQ	7
738	S	PTGWEEPsPESIRRK	14
964	S	NISFSRDsPEENVQS	7
1332	S	NSSTSPAsPPGSIGD	7
1346	S	DGWPRAKsPNGSSSV	7
1378	Y	IDPETDPyVTPGSVI	7
1451	S	SDSKLTWsPGSVTNT	7
1616	S	QSQSLTPsPGWQSLG	6
1691	S	PRGISSPsPINAFLS	6

References indicates that number of articles refereed to phosphorylation sites of TNRC6A. detected by mass spectrometer (references > 5). Orange indicates the amino acids found in our previous study.

**Table 2:** Identified phosphorylation sites of TNRC6A in our previous study.

Amino Acid Position	Motif	Sequence	Modification	Highest pRS Probability (>0.9)
S33		DGLRNsTGLGSQNK	S6(Phospho)	0.990217268
S466	Ago binding domain	VLsNSGWGQTPIK	S3(Phospho)	0.999568224
S486	Ago binding domain	QNTAWDTETsPR	S10(Phospho)	0.999141693
S690	Ago binding domain	SNQSLGWGDSSKPVSsPDWNK	S16(Phospho)	0.990312636
S738	Ago binding domain	EEEPTGWEEPsPESIRR	S11(Phospho)	0.184156224
T791	Ago binding domain	SDQQAQVHQLLtPASAISNK	T12(Phospho)	0.966504514
S797	Ago binding domain	SDQQAQVHQLLTPASAIsNK	S18(Phospho)	0.999999821
S964	UBS-like	QFSNISFSRDsPEENVQSNK	S11(Phospho)	0.917854786
S1119	Q rich	AQVPPPLLsPQVPVSLLK	S9(Phospho)	0.999998212
S1125	Q rich	AQVPPPLLSPQVPVsLLK	S15(Phospho)	0.981702089
T1217	Q rich	QQtPPSQQQPLHQPAMK	T3(Phospho)	0.99997437
T1242	Silencing domain	SFLDNVMPHTtPELQK	T11(Phospho)	0.998789012
S1250	Silencing domain	GPsPINAFSNFPIGLNSNLNVNMDMNSIK	S3(Phospho)	0.997624218
S1332	Silencing domain	LEESPFVPYDFMNSSTSPAsPPGSIGDGWPR	S20(Phospho)	0.998691678
S1346	Silencing domain	AKsPNGSSSVNWPPEFRPGEPWK	S3(Phospho)	0.943672001
S1616	Silencing domain	FFAQSQSLTPsPGWQSLGSSQSR	S11(Phospho)	0.999580026
S1691	Silencing domain	GISSPsPINAFLSVDHLGGGGESM	S6(Phospho)	0.97981894

Amino acid position indicates the position of phosphorylated amino acid candidatae from N-terminal. Motif, domain name of TNRC6A protein. Sequence, peptide fragment in which phosphorylation is detected by mass spectrometry analysis. Modification, phosphoraylated sites in peptide fragment. pRS probability, the probability of phosphorylation.

## Supplementary Materials

The following are available online at https://www.mdpi.com/2073-4425/12/2/271/s1, Figure S1: Effect of phosphorylation of TNRC6A SD fragment on the interaction with RNA silencingrelated factors. Immunoprecipitation using HeLa cells expressing FLAG-HASBP (FHS)-tagged WT TNRC6A-SD fragment, or the fragment with S1332D/S1346D, S1616D/S1691D, S1332D/S1346D/S1616D/S1691D, S1332A/S1346A, S1616A/S1691A, or S1332A/S1346A/S1616A/S1691A was performed using anti-FLAG antibody. CNOT1, CNOT9, and PABP proteins were detected using anti-CNOT1, anti-CNOT9, and anti-PABP antibodies, respectively. α-tubulin was used as negative control. The signal intensities of CNOT1, CNOT9, and PABP proteins were measured using ImageJ, and quantified dividing by the signal intensities by anti-FLAG antibody. The data shown in Figure 3A,C,E and Figure S1A–C were averaged, and means and their standard deviations were shown in Figure 3C,E,F. Figure S2: Effect of phosphorylation of FL TNRC6A on the interaction with RNA silencing-related factors. Immunoprecipitation using HeLa cells expressing FLAGHA-SBP (FHS)-tagged WT TNRC6A-FL, or TNRC6A with S1332D/S1346D, S1616D/S1691D, S1332A/S1346A, or S1616A/S1691A was performed using anti-FLAG antibody. CNOT1, CNOT9, and PABP proteins were detected using anti-CNOT1, anti-CNOT9, and anti-PABP antibodies, respectively. α-tubulin was used as negative control. The signal intensities of CNOT1, CNOT9, and PABP proteins shown in this figure were measured using ImageJ, and quantified dividing by the signal intensities by anti-FLAG antibody. The data shown in Figure 4A,C and this figure were averaged and shown in Figure 4B,D. Figure S3: RNA silencing activity was measured using a luciferase reporter assay. A HeLa cell suspension (1.0 × 10^5^ cells/mL) was inoculated into a well of 24-well culture plates 1 day before transfection. Cells were simultaneously transfected with each 0.5 μg of TNRC6A expression plasmid with 100 ng shRNA against CXCR4 mRNA, 0.5 μg of pGL3-Control (Promega), encoding the firefly luciferase gene, and 0.01 μg of each psiCHECK-CXCR4-MM4, encoding Renilla luciferase gene containing CXCR4 target sites in its 3’UTR, construct by using Lipofectamine 2000 reagent (Thermo Fisher Scientific, Waltham, MA, USA). The transfected cells were lysed with 1× passive lysis buffer (Promega) 48 h after transfection. Luciferase activity was measured using the Dual-Luciferase Reporter Assay System (Promega) and GloMax Discover Microplate Reader (Promega), and the Renilla luciferase activity normalized by firefly luciferase activity (Renilla luciferase activity/firefly luciferase activity) was calculated. Table S1: PCR primers used for mutant expression constructs of TNRC6A.
