# Supplementary material for: Identification of Phosphorylated Amino Acids in Human TNRC6A C-Terminal Region and Their Effects on the Interaction with the CCR4-NOT Complex
Source: Genes (Basel). 2021 Feb 13;12(2):271. doi: 10.3390/genes12020271 (PMC7917804; doi:10.3390/genes12020271)
Supplement: Supplementary file 1 [file genes-12-00271-s001.pdf]

Table S1

**Table S1.** PCR primers used for mutant expression constructs of TNRC6A.

| Primer Name | Sequence(5'→3') |
|---|---|
| TNRC6A-S1346WT-F | GATGGCTGGCCACGTGCCAAATCGCCTAA |
| TNRC6A-S1332WT-R | TCCTATTGAACCTGGAGGACTGGCTGGTG |
| TNRC6A-S1346A-F | GATGGCTGGCCACGTGCCAAAGCTCCTAACGGCTCT |
| TNRC6A-S1332A-R | TCCTATTGAACCTGGAGGAGCGGCTGGTGAAGTACT |
| TNRC6A-S1616A-F | ACCCCTGCTCCCGGCTGGCAGTCTCTC |
| TNRC6A-S1616A-R | CAGAGACTGGCTTTGTGCAAAGAAACG |
| TNRC6A-S1691A-F | AGCAGCCCAGCTCCCATTAACGCTTTTC |
| TNRC6A-S1691A-R | AATTCCTCGGGGGTCGCTGCTGCTTG |
| TNRC6A-S1346D-F | GATGGCTGGCCACGTGCCAAAGATCCTAACGGCTCT |
| TNRC6A-S1332D-R | TCCTATTGAACCTGGAGGATCGGCTGGTGAAGTACT |
| TNRC6A-S1616D-F | ACCCCTGATCCCGGCTGGCAGTCTCTC |
| TNRC6A-S1691D-F | AGCAGCCCAGATCCCATTAACGCTTTTC |

F indicates the forward primer. R, reverse primer.

# Figure S1

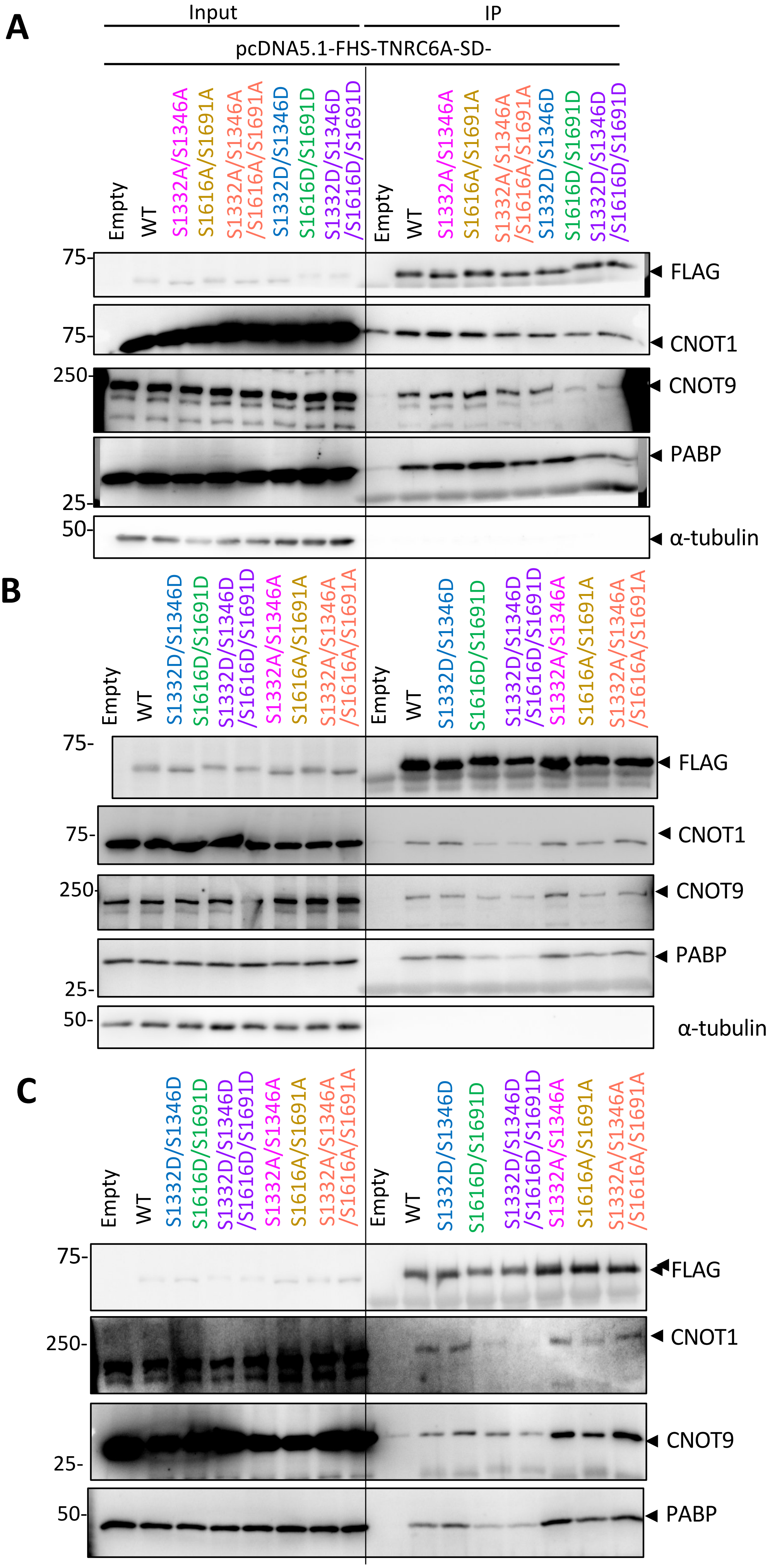


**Figure S1.** Effect of phosphorylation of TNRC6A SD fragment on the interaction with RNA silencing-related factors. Immunoprecipitation using HeLa cells expressing FLAG-HA-SBP(FHS)-tagged WT TNRC6A-SD fragment, or the fragment with S1332D/S1346D, S1616D/S1691D, S1332D/S1346D/S1616D/S1691D, S1332A/S1346A, S1616A/S1691A, or S1332A/S1346A/S1616A/S1691A was performed using anti-FLAG antibody. CNOT1, CNOT9, and PABP proteins were detected using anti-CNOT1, anti-CNOT9, and anti-PABP antibodies, respectively. α-tubulin was used as negative control. The signal intensities of CNOT1, CNOT9, and PABP proteins were measured using ImageJ, and quantified dividing by the signal intensities by anti-FLAG antibody. The data shown in Figure 3A, 3C, and 3E and Figure S1A, S1B, and S1C were averaged, and means and their standard deviations were shown in Figure 3C, 3E, and 3F..

# Figure S2

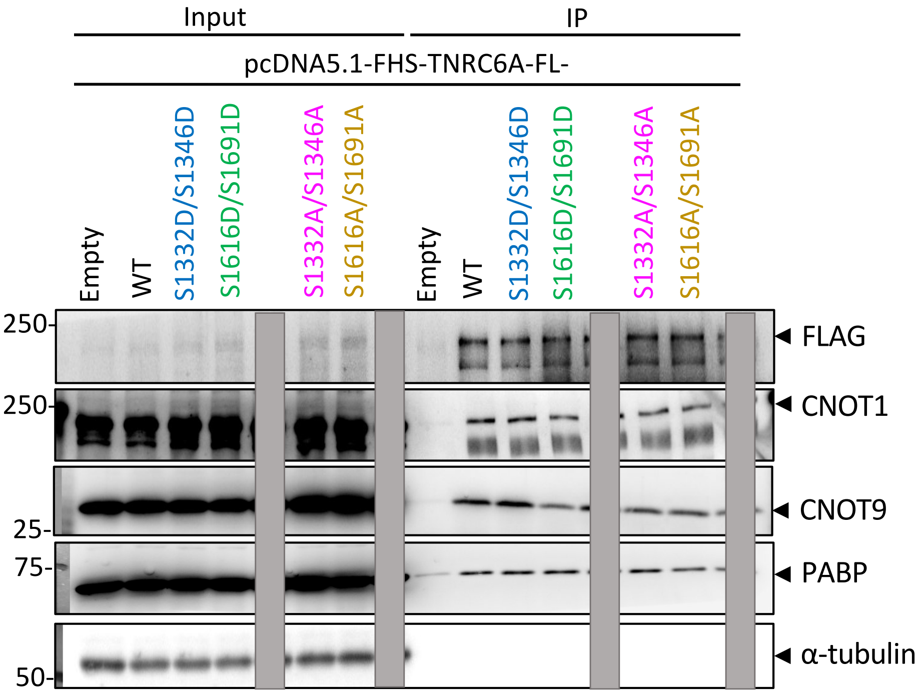


**Figure S2.** Effect of phosphorylation of FL TNRC6A on the interaction with RNA silencing-related factors. Immunoprecipitation using HeLa cells expressing FLAG-HA-SBP(FHS)-tagged WT TNRC6A-FL, or TNRC6A with S1332D/S1346D, S1616D/S1691D, S1332A/S1346A, or S1616A/S1691A was performed using anti-FLAG antibody. CNOT1, CNOT9, and PABP proteins were detected using anti-CNOT1, anti-CNOT9, and anti-PABP antibodies, respectively. α-tubulin was used as negative control. The signal intensities of CNOT1, CNOT9, and PABP proteins shown in this figure were measured using ImageJ, and quantified dividing by the signal intensities by anti-FLAG antibody. The data shown in Figure 4A and 4C and this figure were averaged and shown in Figure 4B and 4D.

# Figure S3

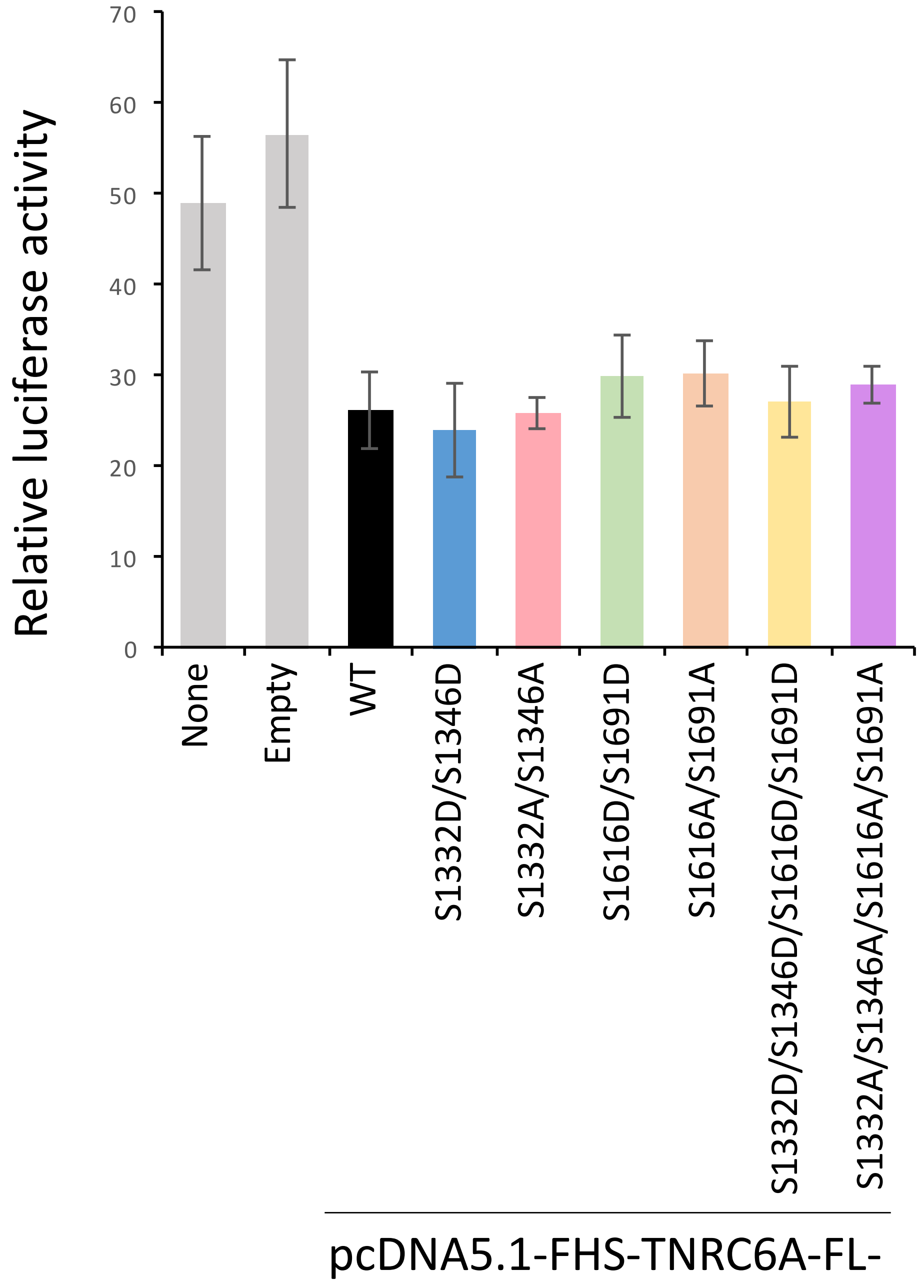


**Figure S3.** RNA silencing activity was measured using a luciferase reporter assay. A HeLa cell suspension (1.0 × $10^5$ cells/mL) was inoculated into a well of 24-well culture plates 1 day before transfection. Cells were simultaneously transfected with each 0.5 µg of TNRC6A expression plasmid with100 ng shRNA against CXCR4 mRNA, 0.5 µg of pGL3-Control (Promega), encoding the firefly *luciferase* gene, and 0.01 µg of each psiCHECK-CXCR4-MM4, encoding *Renilla luciferase* gene containing CXCR4 target sites in its 3'UTR, construct by using Lipofectamine 2000 reagent (Thermo Fisher Scientific, Waltham, MA, USA). The transfected cells were lysed with 1 × passive lysis buffer (Promega) 48 h after transfection. Luciferase activity was measured using the Dual-Luciferase Reporter Assay System (Promega) and GloMax Discover Microplate Reader (Promega), and the *Renilla* luciferase activity normalized by firefly luciferase activity (*Renilla* luciferase activity / firefly luciferase activity) was calculated.
